# Health-seeking behavior and waste management practices among women in major urban markets in Owerri, Nigeria

**DOI:** 10.3934/publichealth.2020015

**Published:** 2020-03-20

**Authors:** Cyprian Ezedike, Eudora Ohazurike, Faisal C Emetumah, Okechukwu O Ajaegbu

**Affiliations:** 1 Department of Geography & Environmental Management, Imo state university, Owerri, Imo state, Nigeria; 2 Department of Political Science, Imo state university, Owerri, Imo state, Nigeria; 3 Department of Sociology, Imo state university, Owerri, Imo state, Nigeria

**Keywords:** health-seeking behavior, health-seeking method, Owerri, market women, sustainable waste management, 3Rs, waste-related diseases

## Abstract

Behavioral patterns on seeking health are pertinent in terms of how waste is managed. However, informal approach towards waste management has led to poor environmental attitude and pernicious health consequences for many Nigerians. Despite plethora of scientific investigation on waste management, there has been paucity of information on health-seeking behavior and waste management practices among market women, hence the need for this research. The study aimed at assessing the health-seeking behavioral pattern of women traders on waste management in major urban markets in Owerri, Nigeria by identifying the extent of their commitment to sustainable waste management practices, investigating health-seeking behaviors that influence their attitude towards waste management and measuring prevalence of waste-related diseases among them. Data collection for the study involved a cross-sectional survey of 739 women trading in three Owerri major urban markets in line with the study's aim. Results show that motivation to manage waste for disease control was effectively predicted by type of trading item (Omnibus Test: χ2 = 13.871, df = 3, p-value = 0.003); Cochran-Armitage tests of trend show that there is no statistically linear trend between the proportions of understanding the 3Rs and the rankings for methods of seeking health; understanding the 3Rs was not determined by health-seeking method as most methods were with motivation to manage waste discordant (4 out 5 health-seeking methods had negative Goodman & Kruskal's *G* values); PCA on the prevalence of waste-related diseases had a two-component structure which followed acute and chronic dimensions; vegetable and plastics comprised the highest waste streams with plastics being most reused waste type while government is mainly responsible for waste disposal. The study recommends a knowledge transfer approach in entrenching sustainable waste management practices.

## 1. Introduction

Waste is any material that is not useful to the current owner and requires getting rid of while waste management involves activities aimed at protecting the overall health and wellbeing of all those who come in contact with waste materials [1]. The way waste is produced, stored, treated and disposed of, affects the overall health condition of any given community [2]. On that note, health-seeking behavior looks at the ability to strive for health care services and also understand the procedural approach to managing illness [3],[4]. The concept of sustainable development as espoused in the Bruntland Report [5],[6] provides the basis for sustainable waste management by ensuring that there is social, economic and environmental balance in handling waste materials. On that note, sustainable waste management covers activities aimed at: reducing, reusing, recycling and recovering energy from waste materials [7],[8]. Much emphasis is placed on waste reduction, reuse and recycling due to their perceived environmental health benefits, hence they are sometimes referred to as “the 3Rs” [9]. However, putting sustainable waste management into practice is a problem [10], as waste disposal practices in Nigeria are still plagued by serious challenges [11]. In Nigeria, waste storage, treatment and disposal does not follow a sustainable approach due to poor supervisory guidelines which makes waste management a free-for-all activity [12]. Furthermore, environmentalism in Nigeria is predominantly anthropocentric in terms of viewpoint [13]. This has resulted to an informal approach to waste with pernicious environmental and health implications for all stakeholders in Nigeria [14]. Similarly, there are serious challenges in terms of domesticating and applying sustainable waste management practices in Nigeria [15]. Moreover, many individuals are not cognizant of the consequences of undiscerning environmental attitude on their health [16]. In addition, health challenges from waste contamination in Nigeria generally stem from carcinogenic heavy metals [17], waterborne diseases [18], respiratory infections, skin diseases [19] as well as spread of disease carrying pests [20]. Studies have been done on health-seeking behavior among communal household members [21], health-seeking behavior in rural areas [22], health-seeking behavior and electronic waste (e-waste) handlers [23]. However, there has been little focus on health-seeking behavior and waste management practices among market women. On that note, the study aims at evaluating the health-seeking behavioral pattern of women trading in three major urban markets in Owerri on waste management practices. Individual objectives were to identify the extent of commitment to sustainable waste management practices by women in selected Owerri urban area markets, to investigate health-seeking behaviors that influence attitude of women towards waste management in selected Owerri urban area markets and to measure prevalence of waste-related diseases among women in selected Owerri urban markets. Literature was reviewed based on these objectives.

### 1.1. Sustainable waste management practices

Sustainable waste management centered on reducing, reusing, recycling or recovering energy from waste has been explicated in many studies [24]–[33]. Nonetheless, understanding sustainable waste management is not wide-spread in many parts of the developing world [34],[35]. In a top Nigerian university campus, recyclables make up over 70% of the waste stream but most of them are not recycled [36]. Similarly, individual daily waste generation in a private higher institution stood at about 0.35 kg/capita/day and material recovery has a market potential of about 1 million US dollars/year which is yet to be realized [37]. The situation is similar in Ghana where sustainable waste management practices covers mainly reusing and recycling metals and Polyethylene Terephthalate (PET) bottles [38]. In terms of waste collection challenges, public education has been identified as the most vital impediment to sustainable waste management [39]. On that note, a knowledge-based approach where a community can be technically informed and practically trained on the positives of sustainable waste management practices has been established and tested in Ibadan Nigeria [40]. In the same vein, a framework for sustainable waste management has been proposed in Egypt which combines improved management systems, government guidelines, increased stakeholder involvement and information sharing [41]. In the United Kingdom, neonatal health workers understand that sustainably managing waste will require more resourcefulness but are ready to make the necessary sacrifice [42].

### 1.2. Health-seeking behaviors that influence attitude

A number of studies have tried to elucidate health issues in Nigeria in terms of health-seeking behavior for mostly common ailments and maternal health afflictions [43]–[53]. However, there are several factors that affect whether or not an individual will seek good health in Nigeria: level of education, socio-economic status, age bracket, location, gender [54]–[56] socio-cultural and religious beliefs [57],[58], traditional customs and marital status [59]–[61]. However, women are more likely to seek health than men [62] while socio-economic factors significantly affect willingness of some Nigerians to engage in sustainable waste management practices [63]. In many Nigerian communities, health-seeking behavior patterns show that low to middle income earners frequented unregistered chemists and pharmacy shops for their health issues, while most high income earners sought the services of private clinics [21],[64]. Similarly, Nigerian university undergraduates depend on their family members, colleagues in health-related disciplines, pharmacies around the campus and trado-religious centers for healthcare information and services [65],[66]. The situation is also similar among Nigerian parents who are mostly reticent in seeking healthcare for their children [67], due to factors like urbanization [68], healthcare quality, communal ethnic diversity [69],[70] and even health insurance uptake [71].

### 1.3. Prevalence of waste-related diseases

In Nigeria, prevalence of acute diseases like malaria, meningitis and other water and air borne diseases are attributable to improper waste management [72]–[74]. Perinatal mortality, birth deformities and leukemia have also been identified as chronic waste-related ailments in many parts of the world [75]. In addition, waste that is contaminated with heavy metals can lead to cardiovascular diseases like heart disease, hypertension [76]–[78], chronic respiratory and reproductive diseases even among children [79],[80]. The situation is not different in Osun State Nigeria where river pollution by heavy metals have high carcinogenic potential [81]. Similarly, lack of vaccination against diseases like tetanus and hepatitis is a significant factor in the health status of informal waste collectors [82]. In the same vein, many cleaners in a Nigerian tertiary hospital are not very knowledgeable about hepatitis infection and vaccination with serious consequences [83]. In addition, Phosphogypsum and phosphate waste from fertilizer production have been identified as radiological with significant carcinogenic effect [84]. This may be why fish sampled from Nigerian waters polluted by agricultural wastes have pernicious levels of organochlorine pesticides [85] with a significant cancer risk. Therefore, it is not surprising that waste materials as a result of oil exploration in the Niger Delta area of Nigeria have been linked to the prevalence of gastrointestinal ailments [86].

## 2. Methods

The study was conducted in Owerri metropolitan area which includes the capital territory of Imo State, Southeast Nigeria. Owerri metropolitan area is approximately 120 km^2^ in area, covering four Local Government Areas (LGAs): Owerri Municipal, Owerri West, Owerri North and Mbaitoli with a 2016 projected population of about 882,500 for the four LGAs [87]. The area lies on the geographical coordinates of latitudes 5°26′ North to 5°53′ North and longitudes 6°97′ East to 7°03′ East. The study which was explorative in approach, focused on three major urban markets in the study area: Eke-Ukwu Owerri market, Owerri relief market and Egbeada modern market. These markets were purposively selected after carefully considering all major markets in the study area; the selected markets were considered largest in terms of size and designation. Cross-sectional survey research design using questionnaire was utilized as data collection instrument. The questions followed the study objectives as the questionnaire was divided into sections covering these objectives. In terms of validation, the questions were designed with contributions by all authors after which they were reviewed and corrections made by the first and second authors. Furthermore, the final questionnaire was also reviewed and affirmed by a female academic with years of experience as a trader in an Owerri major market. The population of the study includes all market women in Owerri metropolitan area who numbered about 35,000 according to market union officials; 780 of these market women were sampled for quantitative data collection. Quota sampling method was used in surveying 260 (780/3) women operating in each of the 3 selected major urban markets in Owerri metropolitan area. Necessary approvals were gotten from the market union prior to commencing the survey. Questionnaires were serially numbered before distribution for identification purposes during data processing. After the field work, 753 questionnaires were retrieved out of which 739 were appropriately filled. Distribution and collection of questionnaires took about 3 months and were concluded by 6^th^ of May, 2019. SPSS version 21 was used in analyzing the study results. In order to facilitate inputting data into SPSS, options for each question were numbered-coded mostly in ascending order; this was done prior to distributing the questionnaires. The 739 retrieved questionnaires were individually inputted into an SPSS data template based on the numbering codes initially given to the respective questions.

### 2.1. Data analysis

Ordinal logistic regression based on proportional odds was used in analyzing data collected on extent of motivation to manage waste in order to control disease (dependent variable) predicted by type of item traded by market women in the study area (independent predictor variable). Firstly, dummy variables which are dichotomous coefficients that can be able to fit into the regression model, were created for both dependent and independent variables. Ordinal logistic regression has assumptions which determine its practicability: proportional odds model should be a good fit [88]; no multi-collinearity for the independent variables indicated by tolerance values of collinearity statistics from dummy variables created [89]; covariate patterns should be acceptable if the independent variable is measured on a scale above nominal [90]. Since the study's regression is the proportional odd model type, Generalized Linear Model (GENLIN) procedure and its Polytomous Universal Model (PLUM) output were used for testing for full likelihood ratio (parallel lines test) [91]. Separate binomial regressions were also carried out for the respective cumulative-dichotomous dependent variables (dummies) so as to confirm the results of the full likelihood ratio test by checking for similarities between the parameter estimates and odds ratio values [92]. Furthermore, negative notations on some of the parameter estimate values do not affect similarities between the values [93]. The thresholds for the dependent variable are the cumulative logits for the dummy variables and are mainly applicable in determining probabilities of the categorical variables [94].

In looking at methods of seeking health and understanding the 3Rs of sustainable waste management, Cochran-Armitage test of trend was used in determining whether there was a linear trend between the levels of each health-seeking method and understanding the 3Rs of sustainable waste management. In doing this, crosstabs were done for each method of seeking health (ordinal) and whether or not market women in Owerri understood the 3Rs of sustainable waste management (dichotomous). In addition, binary logistic regression was ran for each combination of the respective methods of seeking health and understanding sustainable waste management which part of the test procedure [95]. Cases were also weighted by the frequency counts of respective cross-tabulated sets.

Goodman and Kruskal's *G* was used in correlating the extent of motivation to sustainably manage waste so as to have a clean/healthy environment (slight, moderate, considerate and great) and rankings of patronage of different methods of seeking health. In doing this, respective variable cases were weighted by the frequency count for individual crosstabs of the variables under scrutiny. Goodman and Kruskal's *G* ranges from 1 to −1 with positive *G* indicating concordance while negative *G* indicates discordance depending on the significance level [96].

Principal component analysis (PCA) was used in analyzing the prevalence rate of waste-related diseases. PCA was applied because sample size was deemed suitable since it was more than 300. Kaiser-Meyer-Olkin measure of sampling adequacy (KMO) which measures adequacy using scores ranging from 0 to 1; scores closer to 1 are more adequate. In addition, Bartlett's test of sphericity indicates practicability of PCA if its calculated p value is significant. The number of components to be retained are usually determined by eigenvalue scores more than 1, scree plot test, and interpretability criterion using varimax rotation method [97],[98].

## 3. Results

### 3.1. Socio-demographic characteristics and waste management practices among market women that participated in the study

Most of the respondents surveyed were less than 55 years in age with 203 of them aged between 30 and 41 years while 173 were aged between 42 and 53 years. Over 80% of the respondents were educated with 45.9% and 32.9% of them having primary and secondary education respectively, as their highest level of education. In terms of trading item, over 50% of the respondents traded in vegetables/foodstuff while cloths and provisions traders comprised about 17% each. Traders in building materials/electrics comprised 12.6% of the surveyed respondents.

The study results show that vegetable/food and plastics were the commonest waste types with over 80% of the responses (see Table 2). This was followed by paper waste at 11.7% while glass waste had the least responses at 3.6%. Furthermore, government was identified as the main evacuator of waste in major markets in Owerri as 52.8% of the responses were under this category. This was followed by paid disposal (16%) and self-disposal (14.8%). In addition, 9.4% of the respondents disposed their waste by burning it while market unions were responsible for disposing waste as opined by about 7% of the respondents.

**Table 1.** Socio-demographic data on market women who participated in the study (n = 736).

Respondents' characteristics	*N*	%
1. Age groups		
18–29 years	130	20.4
30–41 years	203	31.8
42–53 years	173	27.1
54–65 years	86	13.5
66+	46	7.2
2. Highest level of education		
None	41	5.7
Vocational	90	12.4
Primary	332	45.9
Secondary	238	32.9
Tertiary	22	3.0
3. Type of trading item		
Foodstuff/vegetables	378	52.3
Cloths	125	17.3
Provisions	129	17.8
Building/electric materials	91	12.6

**Table 2.** Common waste type and waste disposal strategies among respondents of the study (n = 736).

	*N*	%
1. Common waste type		
Plastic	182	25.1
Glass	26	3.6
Vegetable	431	59.5
Paper	85	11.7
2. Waste disposal strategies		
Burning	68	9.4
Self-disposal	107	14.8
Union	50	6.9
Government	381	52.8
Paid disposal	115	16

In terms of the extent of waste reuse among women in Owerri major urban markets (see Table 3), the responses indicate that waste reuse was generally not very high. Plastics had the highest responses for great and considerable extent of reuse (cumulatively a little over 35%), while glass and vegetable/food wastes had the most responses for slight extent of reuse at 61.4% and 51% respectively. Reuse of paper and glass waste to great extent had similar responses at about 8% each.

**Table 3.** Extent of waste reuse among market women that participated in the study (n = 736).

Extent of waste reuse	*N*	%
1. Plastic waste		
Great	106	15.1
Considerable	152	21.6
Moderate	174	4.7
Slight	272	38.6
2. Vegetable waste		
Great	67	9.5
Considerable	120	17
Moderate	159	22.5
Slight	360	51
3. Paper waste		
Great	60	8.5
Considerable	130	18.4
Moderate	179	25.4
Slight	336	47.7
4. Glass waste		
Great	62	8.7
Considerable	97	13.7
Moderate	115	16.2
Slight	436	61.4

### 3.2. Ordinal logistic regression model for predicting motivation to manage waste for disease control among respondents of the study

Responses by the study's respondents on the extent of motivation to manage waste in order to control disease (great extent, considerable extent, moderate extent and slight extent) were subjected to ordinal logistic regression based on proportional odds using the categorical types of trading item (foodstuff/vegetables, cloths, provisions and building/electric materials) as the independent predictor variable. The assumptions of ordinal logistic regression were examined for the extent of motivation to manage waste in order to control disease through dummy variables created for the both the dependent and independent variables.

**Table 4.** Test for collinearity tolerance between the predictor and dependent variables for the regression model.

Predictor: type of trading item	Collinearity tolerance coefficients
	Extent of motivation to manage waste for disease control
Vegetable/foodstuff	0.404
Cloths	0.510
Provisions	0.501
Building/electrics*	-

Note: *Reference category.

In terms of collinearity, Table 4 show that all the tolerance values of the types of trading item were above 0.400 for extent of motivation manage waste for disease control, indicating that multi-collinearity was very unlikely. The problem of covariate pattern did not arise for the regression model since one nominal independent variable was used. Therefore, ordinal logistic regression modelling was carried out using the GENLIN procedure in SPSS. Furthermore, PLUM output generated in the GENLIN procedure was used in only carrying out full likelihood ratio test (parallel lines test). The results show that there is an insignificant difference between the proportional odds model and a variant location parameter model (χ2 (6) = 2.284, p = 0.892), indicating that the assumption of proportional odds based on the parallel lines test was met. Separate binomial regressions were also carried out for the respective cumulative-dichotomous dependent variables so as to confirm the results of the full likelihood ratio test. In doing this, the aim was to identify the similarities between the parameter estimates (coefficients) and odds ratio values for each independent variable; similarities are checked since they are likely not going to have exact values in reality.

From Table 5, the results show categories for types of trading item with extent of motivation for disease control were quite similar in the values of their parameter estimates and exponential odds ratio which also agrees with the insignificant result of the full likelihood test using parallel lines.

Table 6 shows that extent of motivation to manage waste in order to control disease-type of trading item regression model fitted the data quite well given that values for both deviance (χ2 (6) = 2.284, p = 0.381) and Pearson (χ2 (6) = 2.257, p = 0.376) in the goodness-of-fit test were insignificant. Moreover, the extent of motivation to manage waste in order to control disease was also predicted significantly by the threshold model as shown by the statistics of the Omnibus test, χ2 (3) = 13.871, p = 0.003. Furthermore, the predictive model based on the type of trading item was effective in predicting the extent of motivation to manage waste in order to control disease as demonstrated by the tests of model effects, Wald χ2 (3) = 13.759, p = 0.003. The value for Akaike's information criterion has also been provided for comparison with other similar models based on the parameters used for this particular model.

**Table 5.** Separate binomial logistic regressions for the three dichotomized variables created for the regression model.

Type of trading item	Extent of motivation for disease control (“great extent”as reference)					
	Parameter estimates(B)			Exp(B) (Odds ratio, OR)		
	Cat1	Cat2	Cat3	Cat1	Cat2	Cat3
Vegetable/foodstuff vs. (ref*)	0.158	0.377	0.105	1.171	1.458	1.111
Cloths vs. (ref*)	−0.307	−0.137	−0.378	0.735	0.872	0.686
Provisions vs. (ref*)	0.529	−0.234	−0.450	0.589	0.792	0.638

Note: Ref* = Building/electrics; Cat1 = Slight vs. other responses; Cat2 = Moderate & considerable vs. other responses; Cat3 = Slight, moderate & considerable vs. other responses.

**Table 6.** Goodness-of-fit tests for the ordinal logistic regression model.

Test	Value	Df	p-value
Deviance	2.284	6	0.381
Pearson Chi-Square	2.257	6	0.376
Akaike's Information Criterion (AIC)	71.951		

Note: Omnibus Test: Likelihood Ratio Chi-Square = 13.871, df = 3, p-value = 0.003; Test of Model Effects: Wald Chi-Square = 13.759, df = 3, p-value = 0.003.

The results in Table 7 shows that odds of respondents selling provisions being motivated to manage waste in order to control disease was 1.524 (95% CI, 0.918 to 2.531) times that of those dealing on building materials while those selling cloths had odds of 1.307 (95% CI, 0.782 to 2.185) times that of those selling building materials. Respondents selling food stuff/vegetables had similar odds to those dealing on building materials since its exponent of .806 is close to 1.

**Table 7.** Parameter estimates for the overall ordinal logistic regression model using GENLIN procedure.

Parameter		B	Std. Error	Sig.	Exp(B)	95% Wald CI for Exp(B)	
						Lower	Upper
Threshold	[Cat1]	0.014	0.2024	0.945	1.014	0.682	1.508
	[Cat2]	0.835	0.2050	0.000	2.304	1.542	3.444
	[Cat3]	2.082	0.2228	0.000	8.020	5.182	12.413
[Vegetable/foodstuff vs. (ref*)]		−0.216	0.2240	0.335	0.806	0.519	1.250
[Cloths vs. (ref*)]		0.268	0.2621	0.307	1.307	0.782	2.185
[Provisions vs. (ref*)]		0.422	0.2587	0.103	1.524	0.918	2.531

Note: Ref* = Building/electrics, Threshold = Extent of motivation to manage waste for disease control, Cat1 = Slight vs. other responses, Cat2 = Moderate & considerable vs. other responses Cat3 = Slight, moderate & considerable vs. other responses, CI = Confidence Interval.

### 3.3. Health-seeking behavior and waste management attitude among market women that participated in the study

Cochran-Armitage test of trend was used in determining whether there was a linear trend between the levels of each health-seeking method and understanding the 3Rs of sustainable waste management. In doing this, crosstabs were done for each method of seeking health (ordinal) and whether or not market women in Owerri understood the 3Rs of sustainable waste management (dichotomous). In addition, binary logistic regression was ran for each combination of the respective methods of seeking health and understanding sustainable waste management. Cases were also weighted by the frequency counts of respective cross-tabulated sets.

**Table 8.** Cochran-Armitage test of trend results for understanding the 3Rs of sustainable waste management and methods of seeking health among respondents of the study (n = 736).

Method of seeking health	Proportions of understanding sustainable waste management's 3Rs for each method of seeking health				CATT score	*p*-value
	Not at all	Not often	Often	Very often		
Divine healer	0.893	0.888	0.820	0.841	3.260	0.071
Chemist shop	0.960	0.915	0.859	0.862	1.783	0.182
Pharmacy	0.870	0.821	0.863	0.890	1.659	0.198
Hospital	0.941	0.874	0.840	0.873	0.799	0.371
Traditional medicine	0.905	0.849	0.837	0.930	0.414	0.520

Note: CATT = Cochran-Armitage test of trend.

The proportions of market women that agreed to understanding the 3Rs based on the rankings for the different methods of seeking health in decreasing order of *p*-values, are shown in Table 8. The results shows that none of the test results specifies a statistically linear trend between the proportions of understanding the 3Rs and the rankings for each method of seeking health.

Goodman and Kruskal's G was used in correlating the extent of motivation to sustainably manage waste so as to have a clean/healthy environment (slight, moderate, considerate and great) and rankings of patronage of different methods of seeking health (see Table 9). In doing this, respective variable cases were weighted by the frequency count for individual crosstabs of the variables under scrutiny.

**Table 9.** Goodman and Kruskal's G scores and significance levels of correlating motivation to manage waste and the different methods of seeking health among respondents in the study (n = 736).

Crosstab variables		Goodman and Kruskal's *G*	*p-*value
Motivation to manage waste so as to have a clean/healthy environment	Divine healer	−0.301	0.000
	Hospital	−0.265	0.000
	Chemist shop	0.159	0.004
	Pharmacy	−0.115	0.039
	Traditional medicine	−0.091	0.062

Results of the Goodman and Kruskal's *G* test showed that all the methods of seeking health were discordant (negative *G*) with motivation to manage waste in order to maintain a clean/healthy environment, except for seeking health in a chemist shop which was concordant (positive *G*). However, the *p* values showed that the concordance and discordance for seeking health through a diving healer, hospitals, chemist shop and pharmacy were significant (*p* ≤ 0.05) while that for seeking health through traditional medicine was not significant (*p* > 0.05).

### 3.4. Analysis of waste-related disease prevalence among respondents of the study using PCA

Results on waste-related disease prevalence were subjected to PCA with the aim of identifying the patterns in the prevalence rate of these diseases. In terms of suitability, the sample size was deemed suitable since it was more than 300 and the calculated Cronbach alpha for the 8 items was 0.816. In addition, Kaiser-Meyer-Olkin measure of sampling adequacy (KMO) was 0.771; individual KMO values ranged between 0.723 and 0.852. Bartlett's test of sphericity was statistically significant, χ2 (28) = 1760.66, *p* < 0.0005. Therefore, both KMO and Bartlett's test results were within acceptable range for conducting PCA [96],[97]. From Table 4, the analysis indicates that only the first two components had > 1 eigenvalues with individual variance explained values of 44.13% and 19.26% respectively.

Rotation of the two extracted components evened values for percentages of variance for the first and second components which were now 32.24% and 31.15% respectively (see Table 10). Furthermore, the scree plot showed viability of retaining two components while the interpretability criterion was also supported by a two-component structure. Therefore, two components were retained with a cumulative variable explained of 63.39%. As shown in Table 5, Varimax rotation was utilized to facilitate a simple structure in line with the interpretability criterion.

**Table 10.** Total variance explained by the PCA for waste-related disease prevalence among respondents of the study.

Component	Initial eigen values			Extraction sums of squared loadings			Rotation sums of squared loadings		

	Total	% of Variance	Cumulative %	Total	% of Variance	Cumulative %	Total	% of Variance	Cumulative %
1	3.530	44.125	44.125	3.530	44.125	44.125	2.579	32.242	32.242
2	1.541	19.264	63.389	1.541	19.264	63.389	2.492	31.147	63.389
3	0.932	11.645	75.034						
4	0.565	7.066	82.099						
5	0.484	6.055	88.154						
6	0.398	4.974	93.129						
7	0.292	3.654	96.783						
8	0.257	3.217	100.000						

Components that loaded strongly to both components are emboldened in Table 11. The rotated component matrix shows that prevalence values of diarrhea and cough are strongly loaded (0.836 and 0.806 respectively) to component 1, while prevalence values of malaria and tetanus are moderately loaded (0.700 and 0.658 respectively) also with component 1. Hypertension and hepatitis are strongly loaded (0.824 and 0.814 respectively) with component 2. The communality values after extraction for the whole components had an average of 0.634 which was relatively adequate.

**Table 11.** Rotated Component Matrix (Varimax rotation) for the extracted components and communalities.

Variable (Disease prevalence)	Components		Communality
	1	2
Prevalence of Diarrhea	0.836	0.131	0.717
Prevalence of Cough	0.806	0.085	0.656
Prevalence of Malaria	0.700	0.026	0.490
Prevalence of Tetanus	0.658	0.373	0.572
Prevalence of Hypertension	0.013	0.824	0.680
Prevalence of Hepatitis	0.055	0.814	0.666
Prevalence of Eczema	0.262	0.798	0.705
Prevalence of Respiratory disease	0.487	0.590	0.585

## 4. Discussion

Study results in Table 2 indicate that most respondents depend on government in terms of waste collection and evacuation. Over dependence on government in terms of waste disposal can be perceived as an informal approach [14] due to inconsistencies and unreliability of government in terms of overall waste management in a Nigerian state as espoused in [11]. Furthermore, burning of waste as practiced by some market women in Owerri demonstrates the challenges of domesticating sustainable waste management in Nigeria [15]. Findings of the study in Table 3 shows that plastic waste had the highest extent of reuse among market women in Owerri. This position is in line with the waste reuse in Ghana were plastics, especially PET bottles have the highest rate of reuse unlike other waste streams [38].

Results of the ordinal regression shows the extent of motivation to manage waste in order to control disease was predicted significantly by the threshold model (Omnibus test, χ2 (3) = 13.871, p = 0.003) which was also supported by the statistically significant test of model effects (Wald χ2 (3) = 13.759, p = 0.003). However, none of these odds had a statistically significant effect in terms of predicting the dependent variable as shown in Table 6. The odds of provision and cloth sellers being high than those of foodstuff/vegetable sellers supports the assertions that sustainable waste management is still rudimentary in a developing country like Nigeria [34],[35]. This is because foodstuff/vegetable sellers would be expected to have more motivation to manage waste in order to control disease since vegetable waste is the commonest waste generated in the study area (see Table 2). This result could be attributable to poor environmental health attitude of many Nigerians [16] which results in many health issues for waste generators and handlers [17]–[20].

The study results on Cochran-Armitage test of trend of the 3Rs of sustainable waste management and methods of seeking health show that understanding the 3Rs is not determined by the extent of seeking health, irrespective of the method as none of the trends were significant (see Table 8). This may be attributable to the anthropocentric perspective of Nigeria's environmentalism [13] which is reflected in the haphazard nature waste is managed in the country [12]. This could be the reason why factors relating to education, socio-economics, location and age have been identified as pertinent in health-seeking behavior [54]–[56]. However, seeking health through a divine healer was very close to linearity with a test score of 3.260 and a significance value of 0.071 which was higher than the significance levels for all other health-seeking methods. This is in agreement with the assertions about the relevance of religious beliefs on the way some Nigerians seek health [58]. In addition, traditional medicine method of seeking health had the lowest test score (0.414) and also showed the most insignificance (p = 0.520). Even though traditional values in Nigeria have been significantly affected by westernization, they are still important in health-seeking behavior [59]–[61].

Results of the study on Goodman and Kruskal's G test of crosstabs between motivation to manage waste so as to have a clean/healthy environment and five different methods of seeking health show that four out of the five methods were not concordant (negative G) with only chemist shop as a health-seeking method have a concordant relationship (positive G). The concordance in seeking health through a chemist shop points towards affordability as many Nigerians patronize these outlets since they may not afford going to hospitals [21],[64]. Similarly, their patronage of conventional health-seeking methods may be detered by urban development and ethnicity [68]–[70]. Furthermore, the Goodman and Kruskal's G test results imply that higher motivation to manage waste in order to have a clean/healthy environment is negatively related with frequently seeking health through divine healer, hospital, pharmacy and traditional medicine. This is because rather than seek health from qualified professionals, some Nigerians resort to family members, quacks and other unorthodox means for healthcare services [65],[66], even for their children [67].

In terms of the PCA carried on the prevalence of eight waste-related diseases, the two component structure interpreted by the PCA was in line with the prevalence of waste-related diseases which follow the main constructs of acute [74],[73] and chronic [76]–[78] waste-related diseases and ailments. Therefore, the prevalence of acute (malaria, diarrhea, cough and tetanus) and chronic (Hypertension, respiratory disease and hepatitis) waste-related diseases can be interpreted as following the PCA structure of component 1 and component 2 respectively. In addition, market women in Owerri especially those dealing on foodstuff/vegetables may be indirectly affected by the pollution in some Nigerian rivers [81] and even fertilizer waste [84] as these pollutants may find their way into food products in the market [85]. Furthermore, the prevalence of these diseases can also be attributed to poor vaccination systems by waste handlers [82] and ignorance about the relationship between these diseases and waste management [83].

## 5. Conclusions

The study elucidated waste management practices of women trading in three Owerri main markets in relation with their health-seeking behavior, attitude as well as waste-related disease prevalence. The study recognized that vegetable and plastics are the most common waste type among the surveyed market women. Furthermore, government was identified as the major evacuator of waste from the markets while plastic waste was the most reused waste stream even though vegetable waste is more common. The study also identified that motivation to manage waste due to disease control was predicted by the type of trading item. In terms of waste management attitude affecting health-seeking behavior, understanding the 3Rs of sustainable waste management was not a significant factor in the method of seeking health among the surveyed market women. Similarly, most methods of seeking health among the market women surveyed were not in agreement with motivation to manage waste in order to have a clean environment which points towards affordable healthcare. Nonetheless, waste-related diseases had acute and chronic diseases as their principal components which implies that it is important to fill the knowledge gap in recognizing the basic nature of these diseases. The study has demonstrated the need for more awareness creation on waste management that adequately considers health attitude in Nigeria, especially among market women who are very resilient micro-economic drivers; they can only be productive when they are in good health. However, domesticating these “alien” principles to many in the developing world must take a practical approach for it to be most effective. Therefore, the study recommends adopting a knowledge transfer approach which involves practical training exercises for health institutions, schools, markets and other public place where various waste streams are continually generated. This will provide more insight into health-seeking behavior and waste management practices, thereby driving home the fundamentals of sustainable development.
